# Supplementary material for: Evaluation of the Antioxidant Activities and Phenolic Profile of Shennongjia Apis cerana Honey through a Comparison with Apis mellifera Honey in China
Source: Molecules. 2023 Apr 6;28(7):3270. doi: 10.3390/molecules28073270 (PMC10097088; doi:10.3390/molecules28073270)
Supplement: Supplementary file 1 [file molecules-28-03270-s001.zip › supplementary Table S1.pdf]

Supplementary Table S1 The LOD, LOQ, linear range and MS characteristics of 13 phenolic compounds and flavonoids.

RT(min)	compounds	LOD (ug/100g)	LOQ (ug/100g)	linear range (ug/100g)	R^2	$[M-H]^{experimental}$	$[M-H]^{calculated}$	error (ppm)	MS/MS
11.68	4-hydroxybenzoic acid	0.15	0.60	0.60-19.20	0.9990	137.0251	137.0244	5.0	93
16.96	vanillic acid	0.40	1.60	1.60-64.00	0.9993	167.0350	167.0350	0.7	152, 123, 108, 91
17.7	caffeic acid	0.06	0.25	0.25-16.00	0.9990	179.0353	179.0350	1.8	135
23.91	p-coumaic acid	0.08	0.32	0.32-12.80	0.9998	163.0406	163.0401	3.3	119, 93
28.77	trans-ferulic acid	0.08	0.32	0.32-25.60	0.9989	193.0507	193.0506	0.3	178, 149, 134
35.98	rutin	0.03	0.10	0.10-16.00	0.9996	609.1420	609.1461	-6.7	301
38.73	trans-cinnamic acid	1.50	6.00	6.00-192.00	0.9992	147.0455	147.0452	2.4	119, 103, 77
39.37	2-cis-4-trans-abscidic acid	0.08	0.40	0.40-12.80	0.9990	263.1282	263.1289	-2.6	219, 204, 163, 152, 139
41.77	quercetin	0.02	0.05	0.05-8.00	0.9998	301.0346	301.0354	-2.6	179, 151
47.15	kaempferol	0.05	0.25	0.25-16.00	0.9996	285.0395	285.0405	-3.4	229, 185, 151
52.43	pinocembrin	0.02	0.08	0.08-6.40	0.9968	255.0660	255.0663	-1.1	213, 171, 151
54.31	chrysin	0.02	0.06	0.06-4.80	0.9997	253.0503	253.0506	-1.3	209, 181, 165, 143
55.38	galangin	0.03	0.13	0.13-8.00	0.9995	269.0453	269.0455	-0.9	213, 169
