# Supplementary material for: Evaluation of the Antioxidant Activities and Phenolic Profile of Shennongjia Apis cerana Honey through a Comparison with Apis mellifera Honey in China
Source: Molecules. 2023 Apr 6;28(7):3270. doi: 10.3390/molecules28073270 (PMC10097088; doi:10.3390/molecules28073270)
Supplement: Supplementary file 1 [file molecules-28-03270-s001.zip › supplementary Table S2.pdf]

Supplementary Table S2 Botanical origin of honey samples identified by melissopalynology analysis

honey Sample	botanical origin
A. cerana	*Toxicodendron vernicifluum* (Stokes) F. A. Barkley (37.55%)
	Cyclobalanopsis glauca (Thunb.) Oerst. (9.15%)
	Actinidia chinensis Planch. (6.73%)
	Spiraea chinensis Maxim. (6.05%)
	Castanea henryi (Skam) Rehd. et Wils. (6.05%)
	Kalopanax septemlobus（Thunb.） Koidz. (4.30%)
	Quercus phillyraeoides A. Gray (4.03%)
	Cyclobalanopsis myrsinifolia (Blume) Oerst. (3.63%)
	Amygdalus persica L. var. persica f.duplex Rehd. (3.50%)
A.m_F	*Hovenia acerba* Thunb. (89.2%)
A.m_p1	*Brassica campestris* L. (32.29%)
	Robinia pseudoacacia L. (17.71%)
	Spiraea chinensis Maxim. (10.41%)
A.m_p2	*Toxicodendron vernicifluum* (Stokes) F. A. Barkley (29.94%)
	Ziziphus jujuba Mill. (25.13%)
	Castanea henryi (Skam) Rehd. et Wils. (9.62%)
	Spiraea chinensis Maxim. (7.48%)
A.m_p3	*Leonurus japonicus* Houttuyn (54.71%)
	Helianthus annuus L. (15.09%)
A.m_p4	*Brassica pekinensis* (Lour.) Rupr. (31.65%)
	Astragalus membranaceus (Fisch.) Bunge. (17.99%)
	Sapium sebiferum (L.) Roxb. (17.99%)
	Spiraea chinensis Maxim. (5.75%)
A.m_p5	*Leonurus japonicus* Houttuyn (43.49%)
	Brassica campestris L. (17.92%)
	Pisum sativum L. (16.03%)
A.m_p6	*Sapium sebiferum* (L.) Roxb. (49.54%)
	Pisum sativum L. (12.84%)
A.m_p7	*Astragalus membranaceus* (Fisch.) Bunge. (57.84%)
	Paulownia fortunei (Seem.) Hemsl. (16.67%)
A.m_p8	*Brassica campestris* L. (57.89%)
	Pisum sativum L. (22.81%)
