# Supplementary material for: Evaluation of the Antioxidant Activities and Phenolic Profile of Shennongjia Apis cerana Honey through a Comparison with Apis mellifera Honey in China
Source: Molecules. 2023 Apr 6;28(7):3270. doi: 10.3390/molecules28073270 (PMC10097088; doi:10.3390/molecules28073270)
Supplement: Supplementary file 1 [file molecules-28-03270-s001.zip › supplementary Table S3.pdf]

Supplementary Table S3 The VIP and *p* values of the marked variables by OPLS-DA analysis

No	variables	VIP	*p* value
1	decenedioic acid	2.908	<0.0001
2	pinobanksin	2.7786	<0.0001
3	pinocembrin	2.3976	<0.0001
4	chrysin	2.0542	<0.0001
5	caffeoylquinic acid isomer 2	1.5173	<0.0001
6	galangin	1.5163	<0.0001
7	caffeoylquinic acid isomer 3	1.4089	<0.0001
8	pinobanksin-5-methyl ether	1.3741	<0.0001
9	UI 2	2.3822	<0.0001
10	UI 1	2.0459	<0.0001
11	isorhamnetin-3-o-neohesperoside	1.3619	0.0004
12	methoxy kaempferol	1.2017	0.0017
13	dimethoxybenzoic acid isomer	1.1781	<0.0001
